# Artificial Intelligence in Bone Fracture Detection: A Review of Evidence, Limitations, and Clinical Integration

**DOI:** 10.7759/cureus.97674

**Published:** 2025-11-24

**Authors:** Ahmed Elkohail, Ali Soffar, Ashis Paul, Larisa Radu, Mohamed Wasim Shaffe Ahamed, Ahmed Swealem, Aqil Ahamed Mohideen Ahamed Sha, Haritha Haridas Mandoth Veetil, Mohammed S Millat, Rhia Shah

**Affiliations:** 1 Orthopedics and Traumatology, Princess Royal University Hospital, King’s College Hospital NHS Foundation Trust, London, GBR; 2 Orthopedics, Southmead Hospital, North Bristol NHS Trust, Bristol, GBR

**Keywords:** ai, artificial intelligence, computed tomography (ct), deep learning, fracture detection, magnetic resonance imaging (mri), musculoskeletal imaging, radiography

## Abstract

Medical imaging is rapidly being improved by artificial intelligence (AI), with deep-learning systems performing well in radiography, CT, and MRI for fracture detection, classification, and localization. This narrative review examined recent evidence spanning different types of bone fractures, alongside soft-tissue injuries relevant to orthopedic decision-making. Across multiple meta-analyses and external validations, reported sensitivities and specificities commonly range from 0.85 to 0.95, while AI also supports workflow triage and reader confidence. Persistent gaps include limited generalizability, inconsistent reference standards, spectrum bias, regulatory and ethical challenges, and implementation costs. We outline pragmatic quality considerations and emphasize prospective, multi-center trials and transparent reporting for safe clinical integration. AI should augment clinicians, improving speed, accuracy, and overall patient outcomes.

## Introduction and background

Artificial intelligence (AI), introduced by John McCarthy in 1956, seeks to replicate human intelligence through computers, with machine learning (ML) as a subset that improves through experience [1]. The utilization of AI technologies is proliferating across various disciplines, and medicine has emerged as a key domain for its application and innovation [2]. By 2026, AI is expected to save approximately $150 billion annually in the U.S. healthcare, primarily by enabling proactive care, automated data review, and individualized risk profiling that detects treatable diseases earlier, resulting in fewer hospitalizations, clinic visits, and treatments [2,3].

Over the last decade, AI applications in orthopedics have primarily focused on diagnostics, particularly image interpretation [4,5]. AI supports them by enhancing diagnostic accuracy, reducing errors, and minimizing fatigue. Algorithms have been utilized in diagnosing fractures, osteoarthritis, assessing bone age, and evaluating bone strength [6].

Many studies show that convolutional neural networks (CNNs) can detect a range of upper- and lower-limb fractures on X-rays, including hip, calcaneal, and radial injuries, with reported accuracies up to 98% and performance that matches or even surpasses that of human readers [6-11]. Recent systematic reviews have shown that AI models, particularly CNNs, achieve high diagnostic accuracy, with sensitivity and specificity rates ranging from 81 to 94% and 83 to 92%, respectively [12].

AI applications encompass tasks, such as fracture detection, classification, segmentation, and workflow optimization [13]. Studies consistently report that AI performance equals or exceeds human radiologists in fracture diagnosis [14-16]. However, significant limitations persist, including inadequate reference standards, regulatory challenges, external factors like casts affecting accuracy, and the need for human validation [17,18]. In this review, we aimed to highlight the literature on the use of AI in orthopedic fracture detection.

## Review

Methodology

This review was conducted as a narrative synthesis to map and contextualize the evidence on AI for bone fracture detection across imaging modalities and anatomical regions. We prioritized breadth and interpretive integration over exhaustive, protocol-driven aggregation, aiming to clarify where AI adds diagnostic value, where limitations persist, and how clinical integration is unfolding.

This review used targeted searches of PubMed/MEDLINE, Scopus, and Google Scholar, combining musculoskeletal and AI terms and snowballing key reviews to capture primary studies on fracture detection, classification, localization, and segmentation. We included original research, validation studies, or quantitative reviews on radiography, CT, or MRI that reported diagnostic metrics and provided enough methodological detail to assess datasets and reference standards. Populations spanned pediatric to geriatric, including acute and occult fractures across upper and lower limbs, skull/facial bones, and spine.

Data extracted per study included modality, anatomical target, dataset size/provenance, ground truth (radiologist consensus, surgical confirmation, CT/MRI adjudication), model architecture/training (augmentation, transfer learning), validation strategy (internal/external/temporal), and performance metrics; region-level metrics (IoU/Dice, box AP) were recorded for localization/segmentation. Evidence was organized by anatomical region with a parallel strand on soft-tissue injuries. Quality appraisal followed clinical AI best practices, emphasizing dataset representativeness, leakage avoidance, robust standards, external validation, and transparency, with attention to spectrum bias, class imbalance, and cross-site/device drift. Findings were narratively integrated, contrasting models with human readers, highlighting externally validated/prospective designs, and foregrounding miss rates, reader-aid effects, triage utility, workflow fit, explainability, and turnaround time.

Artificial intelligence in lower-limb fracture detection

Femoral Neck Fracture Detection

AI offers advantages, such as early fracture detection, timely initiation of treatment, reduced postoperative recovery periods, and avoidance of additional costs associated with misdiagnosis [5]. Femoral fractures represent a significant health concern among the elderly [7,19]. The incidence is expected to double over the next three decades, coinciding with the worldwide rise in the aging population [20,21].

A retrospective study by Beyaz et al. applied a convolutional neural network (CNN) to detect femoral neck fractures from pelvic X-rays. The dataset of 234 images from 65 patients was expanded to 2,106 using data augmentation. The CNN, comprising five convolutional blocks with batch normalization, ReLU activation, pooling, dropout, and fully connected layers, was optimized with a genetic algorithm (GA). Using five-fold cross-validation, the best results were obtained with 50×50 pixel images, achieving 83% sensitivity, 73% specificity, and 77.7% accuracy, which improved to 79.3% with GA optimization. The study highlighted deep learning’s potential for fracture detection and the benefits of smaller image sizes for accuracy and computational efficiency in limited datasets [7].

**Figure 1:**
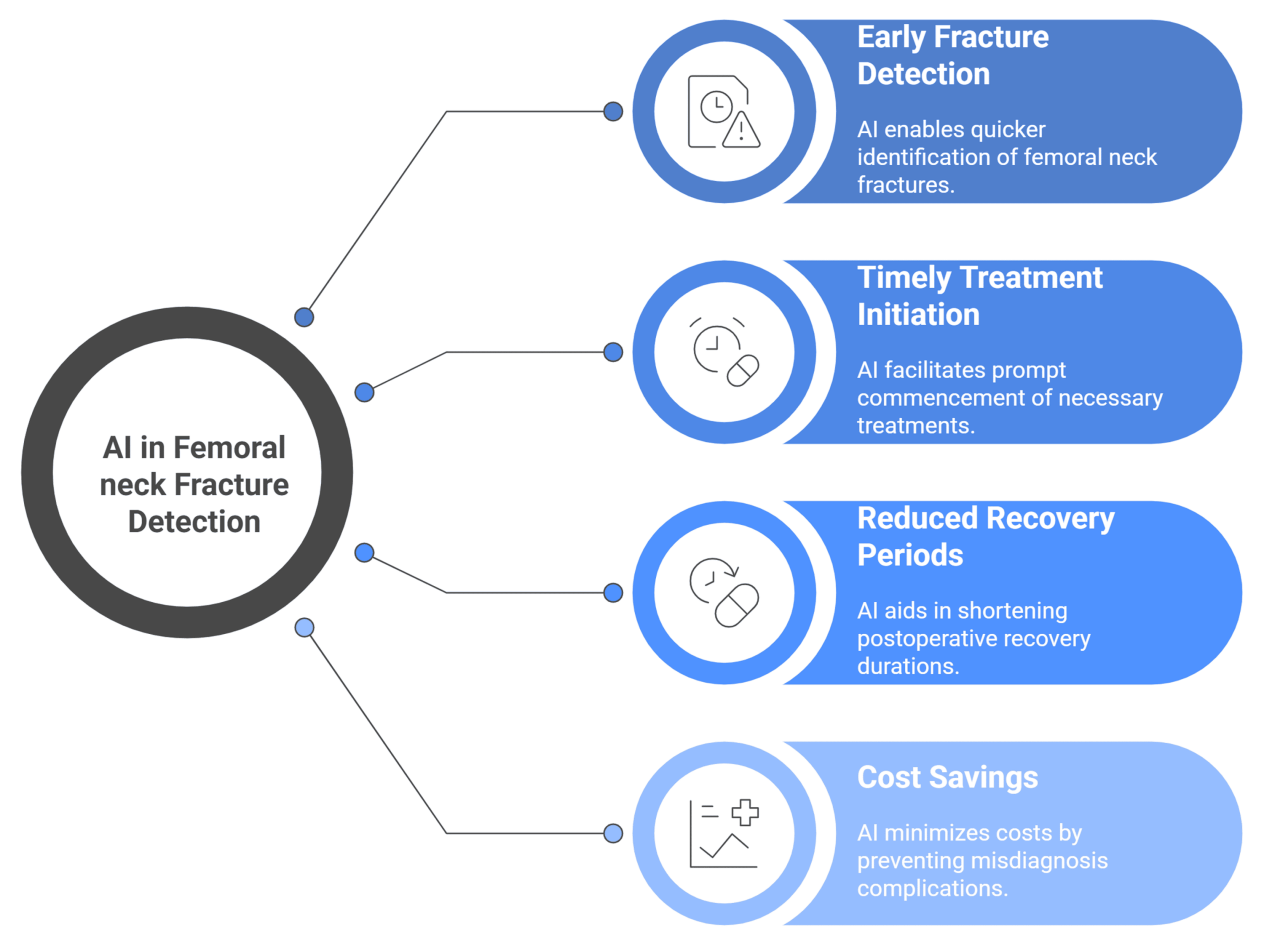
AI in femoral neck fracture detection. This image, created by the authors of this study, summarizes the use of AI in femoral neck fracture detection, based on references [20-24]. AI: artificial intelligence

A recent meta-analysis found that AI-assisted radiological evaluation of femoral neck fractures achieved 91% accuracy, 87% sensitivity, and 91% specificity, demonstrating human-comparable performance and reliable clinical applicability in radiographic fracture assessment [22]. This meta-analysis of AI-assisted radiological evaluation of femoral neck fractures produced findings consistent with those of recent meta-analyses on AI-based fracture detection [12,22-24]. The 2022 meta-analysis of 42 research studies by Kuo et al. reported a sensitivity of 92% and specificity of 91% for general fractures [23]. In the same year, Zhang et al.’s meta-analysis of 39 studies showed 96% accuracy, 90% sensitivity, and 92% specificity [24]. In 2023, Lex et al.'s meta-analysis of 39 studies reported 79% accuracy, 89.3% sensitivity, and 87.5% specificity for hip fractures [25]. Most recently, Jung et al.’s 2024 meta-analysis of 66 studies demonstrated 91% accuracy, 92% sensitivity, and 90% specificity for hip fracture detection using AI (Figure 1) [12].

Hip Fracture Detection

In Cheng et al.'s analysis, 25,505 images were used to train the AI model that was used to identify hip fractures on anteroposterior pelvic radiographs. They reported 91% accuracy, 98% sensitivity, a 2% false-negative rate, and an AUC of 0.98 using gradient-weighted class activation mapping for validation. The visualization algorithm demonstrated the model's strong diagnostic performance by achieving 95.9% accuracy in lesion localization [26]. AI models successfully supported hip fracture diagnosis and outcome prediction, according to a recent systematic review and meta-analysis of 39 studies. Using over 39,000 radiographs and 714,000 clinical cases, AI achieved a sensitivity of 89.3% and a specificity of 87.5%, with a strong mortality prediction (AUC 0.84). AI diagnostic accuracy was comparable to that of radiologists and surgeons, showing promising clinical applications [25].

Another study developed a convolutional neural network (Mask R-CNN) to classify, localize, and segment ankle fractures using 326 fibula fractures and 423 non-fracture radiographs. On internal and external validation sets, diagnostic accuracy reached 89-90% with AUCs of 0.93-0.99 across Weber classes. Segmentation achieved mean intersection over union (IoU) scores of 0.65 for bounding boxes and 0.47 for fracture morphology, demonstrating robust classification and promising external validity [27].

Artificial intelligence in upper limb fracture detection

CNNs matched subspecialist performance and even surpassed general orthopedic surgeons in identifying and grading proximal humeral fractures [16,28]. The Choi et al. study developed a dual-input CNN using both anteroposterior (AP) and lateral elbow radiographs to detect pediatric supracondylar fractures. Trained on 1,266 image pairs, the model achieved areas under the receiver operating characteristic curve (AUCs) of 0.976-0.992 across validation and external tests. Diagnostic performance was comparable to radiologists, with high sensitivity (93.9-100%) and negative predictive value (NPV) (97.8-100%), though specificity and positive predictive value (PPV) were slightly lower. Results highlight its feasibility and potential to support accurate pediatric fracture diagnosis in clinical practice [29].

Olczak et al. conducted a study utilizing distal radius radiographs to evaluate the performance of their detection model. Their analysis demonstrated that the model achieved a fracture detection rate of 83%, highlighting the potential role of artificial intelligence in improving the diagnostic accuracy of radial fractures [16]. Another study used AI to detect radius and ulnar styloid fractures on X-rays. Using augmented datasets, it achieved 98% and 91.1% accuracy, with AUCs of 0.991 and 0.956, demonstrating high diagnostic performance [30].

Kim and MacKinnon evaluated deep-learning algorithms using wrist radiographs, including both fracture and non-fracture cases. Their model achieved a sensitivity of 0.90 and a specificity of 0.88. They emphasized the technique’s transferability, suggesting broad applications in medical imaging with the potential to enhance workflow efficiency and reduce clinical risks [31]. Raisuddin et al. developed and evaluated DeepWrist, a deep-learning pipeline for distal radius fracture detection. Using conventional radiographs, performance was excellent on a general test set (average precision 0.99; AUC 0.99) but significantly lower on challenging CT-confirmed cases (average precision 0.64; AUC 0.84). Findings emphasize careful evaluation of artificial intelligence models in difficult scenarios and the necessity of robust testing before clinical application [32]. The Yang et al. study proposed a two-stage convolutional neural network for scaphoid fracture detection. Faster R-CNN isolated scaphoid bones, followed by ResNet-based feature extraction with feature pyramid network (FPN) and Convolutional Block Attention Module (CBAM) for detection and classification. The model achieved high accuracy (99.7% for bone detection, 0.853 for fracture detection) and strong diagnostic metrics, showing promising clinical potential [33].

Artificial intelligence in skull and spinal fractures detection

Skull Fractures

Using CT scans, Yang et al. developed a deep-learning model based on the feature pyramid network (FPN) to detect a broken nose. The model achieved 0.8478 sensitivity and 0.8667 specificity. Diagnostic performance was compared with radiologists and physicians, showing that AI-assisted reading significantly enhanced their accuracy in detecting nasal bone fractures [34]. A hybrid deep-learning pipeline (YOLOv4 {Seattle, WA: University of Washington} for one-stage detection and ResUNet++ {Dhaka, Bangladesh: BUET} for segmentation) accurately identified skull fractures on CT. In facial regions, it reached a sensitivity of 0.81, with consistently strong results across most bones; performance was weaker for nasal fractures, likely due to the small sample size [35].

Wang et al. evaluated CNN (U-Net {Freiburg, Germany: University of Freiburg} and ResNet {Beijing, China: Microsoft Research}) for detecting and classifying mandibular fractures on spiral CT scans from 686 patients. The models achieved high performance, with U-Net showing a DICE of 0.943, regional accuracies above 90%, and a mean AUC of 0.956. CNNs demonstrated reliable accuracy, supporting their role in improving diagnostic efficiency and access [36]. In order to train CNN-based models (DenseNet-169, ResNet-50, Faster R-CNN {Beijing, China: Microsoft Research}, YOLOv5) for the detection of mandibular fractures, another study retrospectively examined 1,710 panoramic radiographs. Models outperformed experts with near-perfect classification (AUC 100%) and high detection accuracy, indicating significant clinical utility in supporting clinician performance to expert-level standards [37]. For instance, some researchers trained a YOLOv5 model on panoramic radiographs to detect and classify mandibular fractures across six anatomical regions, achieving high accuracy (up to 96%), strong AUCs (≈0.94), and promising diagnostic aid potential [38].

Vertebral Body Fractures

AI has also been applied to the detection of vertebral body compression fractures on CT imaging. Roth et al. demonstrated the feasibility of using a CNN for automated identification of posterior element fractures, achieving an AUC of 0.857 with sensitivities of 71% and 81%, despite relying on a relatively small dataset. In subsequent work, the same group developed an automated machine-learning (ML) system employing support vector machine regression (SVMR) to detect, localize, and classify thoracic and lumbar vertebral compression fractures, reporting a sensitivity of 95.7% and a false-positive rate of 0.29 per patient [6].

**Figure 2:**
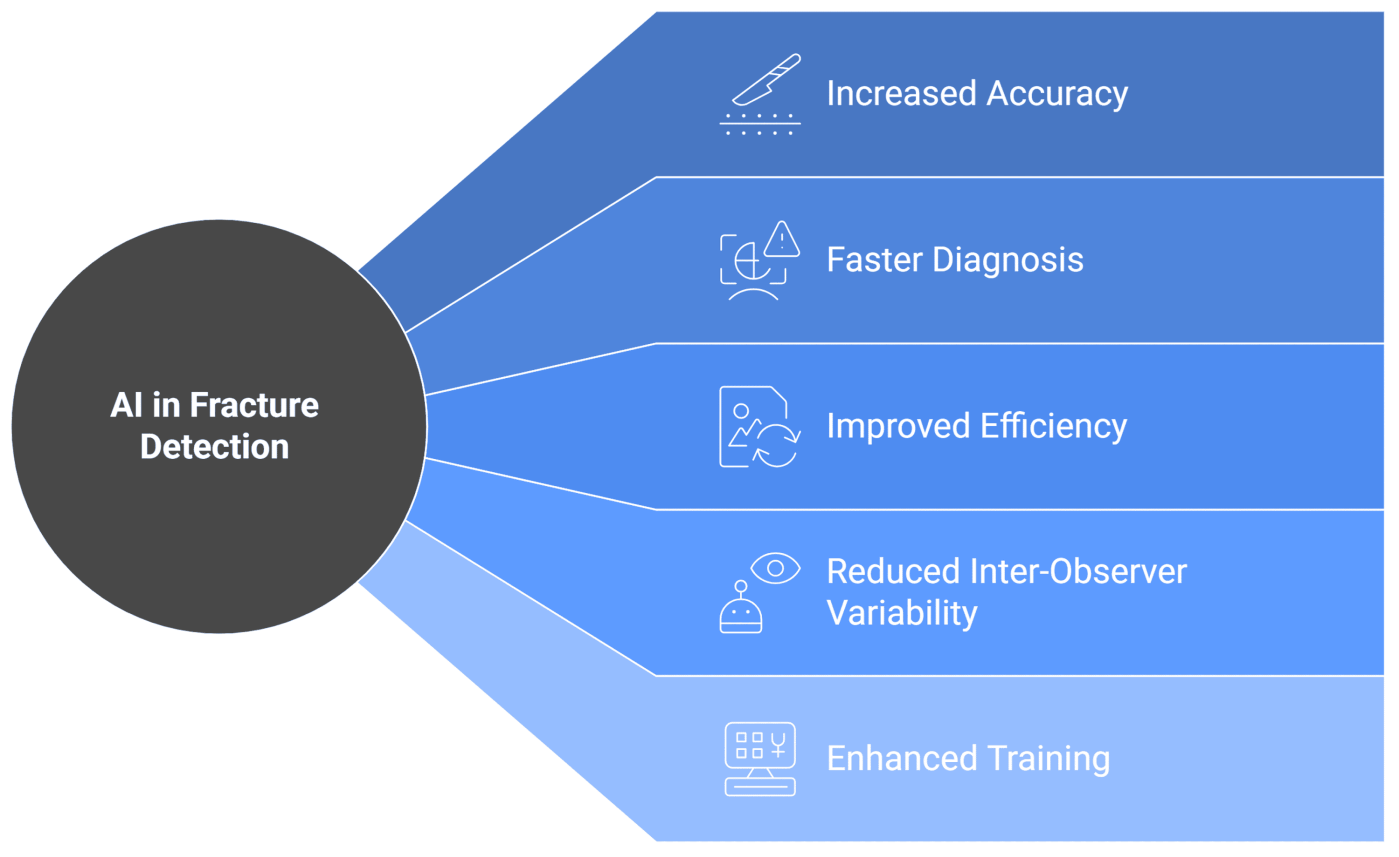
Benefits of using AI in orthopedic fracture detection. This image, created by the authors of this study, summarizes the AI benefits in orthopedic fracture detection, based on references [19-41].

Nicolaes et al. conducted a retrospective study to externally validate a CNN model for detecting vertebral fractures in 4,810 CT scans from Chinese patients aged ≥50 years. The algorithm achieved an area under the receiver operating characteristic curve (AUROC) of 0.94, with 94% sensitivity and 93% specificity, demonstrating excellent accuracy in identifying moderate-to-severe VFs and supporting its role as a radiology aid [39]. Li et al. evaluated ResNet50 deep learning on CT scans from 433 patients (296 with malignant vertebral fractures, 137 with benign vertebral fractures), achieving per-patient sensitivity of 95%, specificity of 80%, and accuracy of 88%. Deep learning showed strong potential for distinguishing benign vs. malignant vertebral fractures [40]. In Chen et al.'s study, a deep-learning model was developed to distinguish fresh from old vertebral compression fractures using digital radiographs, with MRI as reference. In 1,099 patients, the ensemble model achieved AUC 0.80, accuracy 74%, sensitivity 80%, and specificity 68%, with superior performance in lateral views and severe fractures (Figure 2) [41].

Soft-tissue and orthopedic sports injuries

Nearly half of all reported musculoskeletal injuries are associated with the knee, underscoring its vulnerability as one of the most commonly affected joints in both sports-related and everyday trauma [42]. Among these injuries, anterior cruciate ligament (ACL) tears are particularly common, with noncontact mechanisms accounting for up to 78% of all sports-related knee injuries [43]. AI can accurately predict patients at risk of primary or recurrent ACL injury, assist with intraoperative identification of complex anatomical landmarks, and optimize postoperative pain management and rehabilitation strategies [8,44-47].

Chang et al. developed a CNN-based model to detect ACL tears. This model first cropped MRI scans to locate the ACL before identifying the tears. The authors tested model accuracy using one, three, and five knee slices and found that adding more slices boosted performance. With five slices, the model reached a sensitivity of 0.940 and a specificity of 0.890 [48]. Liu et al. used a two-CNN pipeline on knee MRI to localize the ACL and detect injuries, with arthroscopy as the reference. The model’s performance sensitivity of 0.96 was statistically indistinguishable from that of five radiologists, indicating that ML can handle complex ACL diagnostic tasks [49]. Štajduhar et al. created a support vector machine (SVM) to identify both mild and complete ACL tears. His findings highlight the potential of computer-aided decision-making to differentiate between complete and partial tears [50].

Xie et al. used a CNN to enhance MRI quality for tibial plateau fractures with concomitant meniscal injuries. Compared with arthroscopic findings, the model achieved a sensitivity of 96.9%, a specificity of 93.2%, and an accuracy of 95.3%. The clearer, AI-enhanced images yielded diagnoses that matched intraoperative assessments [51,52]. CNNs for osteoporosis fracture recognition have been developed to directly evaluate bone mineral density from radiographs [36,37].

Limitations and future directions

AI requires substantial capital investment and imposes significant financial strain on healthcare systems, which could ultimately limit its widespread adoption and hinder the seamless integration of these technologies into routine clinical practice [5,53]. Applying AI models beyond the datasets or institutions in which they were developed (external validity) requires careful evaluation. Systematic algorithmic errors may result in harmful, large-scale consequences for patients. Therefore, rigorous methods for model design and validation, grounded in established principles, are essential before introducing AI into clinical practice. To minimize these risks, AI should serve as a supportive tool in clinical decision-making rather than a replacement. Clinicians must remain aware of its limitations and exercise caution until external validity is demonstrated within acceptable error margins [15,54].

Challenges also include the time required for AI implementation (during both preparation and intraoperative use), inconsistent reliability of current technologies, and the lack of long-term outcome studies. Consequently, reducing the cost and time associated with AI applications and conducting extended follow-up research are essential. Additionally, ethical concerns arise with the use of ML in orthopedic surgery. Handling large datasets increases the risk of violating patient confidentiality and consent, particularly when conflicts between patient welfare and commercial interests are present, unless strict safeguards are ensured [5].

Carmo et al. underscore that most CNN studies on fracture detection lack external validity. In their large systematic review, only four studies, about 11% of those identified, demonstrated temporal and geographic generalizability beyond a single hospital. They advocate adopting standardized reporting to establish a reliable ground truth for fracture-recognition models, such as using the Clinical Artificial Intelligence Research (CAIR) checklist [55,56].

Future Directions

The integration of AI into radiological diagnosis has become increasingly evident among medical practitioners and in daily clinical routine. In orthopedics and traumatology, AI serves as an important tool for identifying many conditions, including fractures, dislocations, and bone lesions [15]. Initial assessment of trauma, such as distal radius fractures, is usually carried out by medical interns or emergency physicians. Accurate diagnosis followed by proper immobilization with a cast or splint can help prevent fracture displacement. Therefore, early diagnosis and management are crucial [57].

While AI-powered image diagnosis is expected to advance considerably, it's crucial to remember that it serves only as a supplementary tool. A comprehensive diagnosis requires evaluating other clinical data, including patient histories, physical examinations, and blood tests. To bolster the reliability of AI-based orthopedic injury diagnostics and predictions, further cohort studies and large randomized controlled trials are essential [57,58].

**Figure 3:**
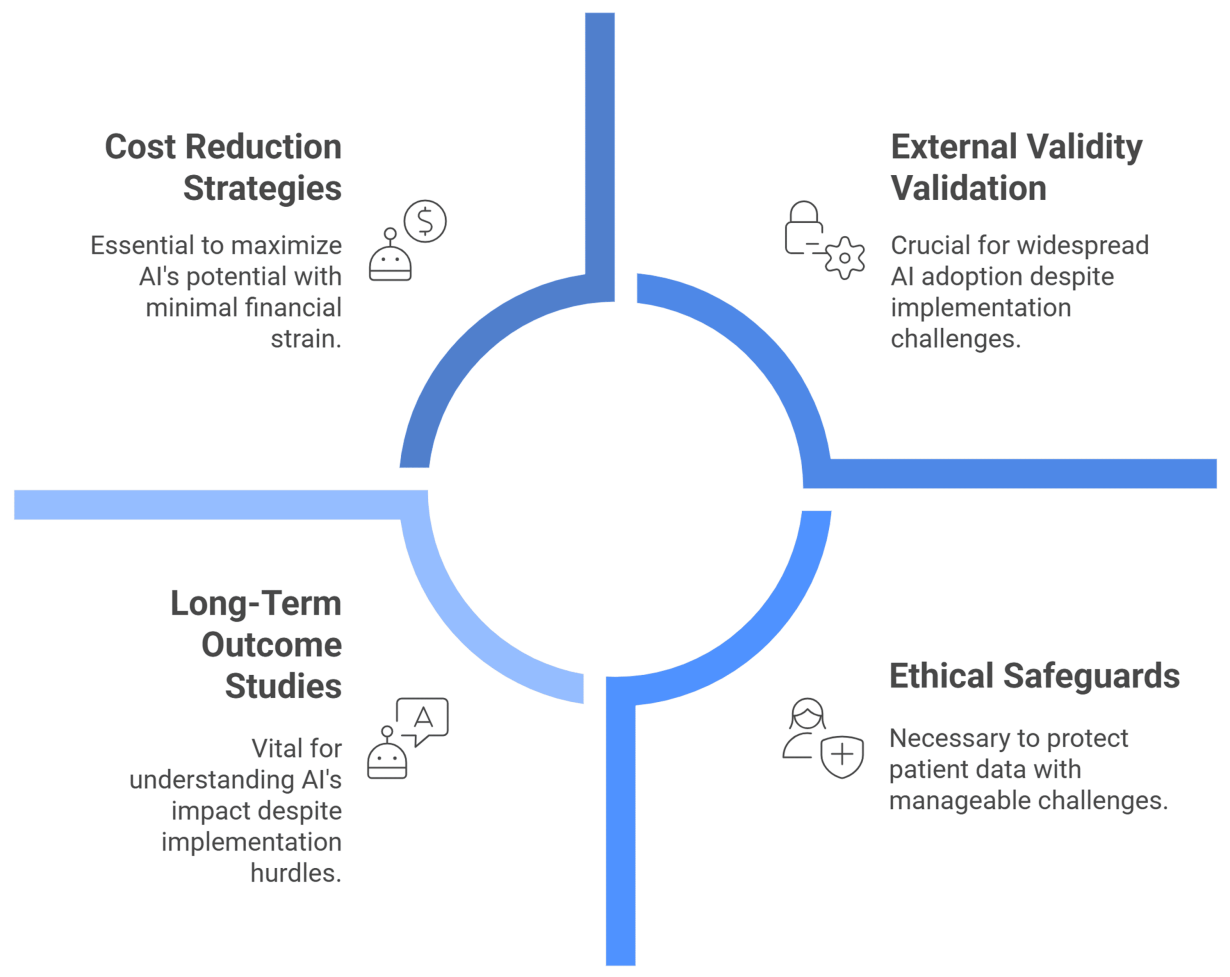
Limitations and future directions. This image, created by the authors of this study, summarizes the limitations and future directions, based on references [53-59].

Future research needs to focus on externally validating algorithms in real-world clinical settings, including prospective randomized clinical trials. This is a crucial step for clinical deployment and should involve a fair comparison with clinicians, providing them with routine clinical details. Currently, AI serves as a diagnostic adjunct, enhancing workflow by screening or prioritizing images and highlighting areas of interest for radiologists. It can also boost diagnostic certainty by acting as a "second reader" or providing an interim report before official radiologist interpretation (Figure 3) [23].

AI and medical imaging diagnosis are deeply integrated. AI has significantly enhanced the precision of medical imaging diagnosis through technological improvement. Conversely, the needs of medical imaging continuously drive innovation in AI technology. While AI is not yet widely adopted in clinical imaging diagnosis, its promising future is undeniable [59].

## Conclusions

AI has demonstrated remarkable potential in enhancing bone fracture detection, often achieving accuracy comparable to or exceeding that of human experts. Its ability to improve diagnostic speed, reduce errors, and support clinical workflows highlights its value as a supplementary tool. However, limitations related to external validation, ethical considerations, and financial costs emphasize the importance of cautious, evidence-based adoption.

Future integration of AI in orthopedics requires rigorous validation through large-scale, multi-institutional trials to ensure reliability across diverse populations and imaging conditions. AI should be positioned as a supportive aid rather than a replacement, complementing radiologists by streamlining workflows and improving diagnostic certainty. With continued research and responsible implementation, AI can significantly advance orthopedic care and patient outcomes.
